# Circadian disruption and circulating TGF-β1 in workers: evidence from Mendelian randomization and observational data

**DOI:** 10.3389/fimmu.2026.1904019

**Published:** 2026-09-10

**Authors:** Seunghyun Lee, Xiaoxue Ma, Youjin Kim, Hua Zhu, Wanhyung Lee

**Affiliations:** 1 Department of Convergence Medicine, School of Medicine, Pusan National University, Yangsan, Republic of Korea; 2 Department of Pediatrics, The First Hospital of China Medical University, Shenyang, China; 3 Department of Neurology, Asan Medical Center, Seoul, Republic of Korea; 4 Department of Preventive Medicine, College of Medicine, Chung-Ang University, Seoul, Republic of Korea

**Keywords:** shift work, Mendelian randomization, biomonitoring, immune dysregulation, circadian rhythm, TGF-β1

## Abstract

**Background:**

Circadian disruption is increasingly recognized as an important biological stressor underlying chronic disease, yet its direct immunological effects remain unclear. Observational studies are limited by residual confounding and reverse causation.

**Methods:**

We integrated two-sample Mendelian randomization (MR) with an exploratory observational biomarker study. Genetic instruments for genetically proxied shift-work propensity were derived from the UK Biobank GWAS, and outcome data for 46 circulating proteins were obtained from the AGES–Reykjavik Study in Iceland. Inverse-variance weighted (IVW) was primary estimator, with four additional MR estimators and sensitivity analyses assessed consistency and potential violations of MR assumptions. False discovery rate (FDR) correction was applied across the 46 primary IVW tests. We also measured eight circulating immune mediators among 61 Korean workers to obtain complementary observational evidence relevant to the MR findings.

**Results:**

MR demonstrated that genetic predisposition to shift work causally increases circulating TGF-β1 (β=1.15, 95% CI 0.28-2.02) and SMAD4 (β=1.12, 95% CI 0.20-2.05), with no evidence of heterogeneity, horizontal pleiotropy, or reverse causation. MR-PRESSO detected no outlier variants. In the observational analysis, shift workers exhibited higher serum TGF-β1, providing consistent supportive evidence, while pro-inflammatory cytokines measured in EDTA plasma showed no consistent differences.

**Conclusions:**

Circulating TGF-β1 emerged as a candidate immune signal associated with genetically proxied shift-work propensity. However, neither association survived FDR correction, SMAD2 and SMAD3 estimates were discordant, and the observational association attenuated after age adjustment. Further longitudinal studies are needed to clarify biological and mechanistic relevance.

## 1 Introduction

Circadian rhythms coordinate metabolism, endocrine signaling, and immune function, and their disruption has been implicated in cardiometabolic, psychiatric, and sleep disorders (1–3). Many of these outcomes share common pathways of low-grade inflammation and immune dysregulation (4, 5). Night and rotating shift work are major sources of circadian misalignment, but they also reflect a broader propensity to engage in nonstandard work schedules, shaped by occupational, behavioral, and tolerance factors (6, 7). In epidemiological studies, this complexity has contributed to coarse exposure assessment and inconsistent evidence regarding systemic inflammatory changes in shift workers (8–11). Whether genetic liability to shift-work employment, as a proxy for long-term circadian disruption, influences circulating immune mediators has not been established (12, 13). In particular, it remains unclear whether circulating cytokines and cytokine signaling proteins are associated with genetic liability to shift work.

Mendelian randomization (MR) offers an opportunity to overcome these limitations by using genetic variants as instrumental variables for shift-work propensity (14). Because validated genomic instruments that directly capture circadian rhythmicity or misalignment are still scarce, current MR analyses must rely on shift-work–related variants as a practical proxy (15, 16). To date, no MR study has evaluated the association of such genetically anchored circadian disruption on circulating immune markers.

We therefore combined a two sample MR analysis of genetically proxied shift work propensity and 46 targeted serum protein outcomes spanning cytokines and cytokine signaling proteins with an exploratory observational biomarker study in Korean workers. We aimed to evaluate potential causal associations between shift work propensity and circulating immune proteins and to obtain complementary observational evidence.

## 2 Materials and methods

### 2.1 Mendelian randomization

#### 2.1.1 Study design

**Figure 1:**
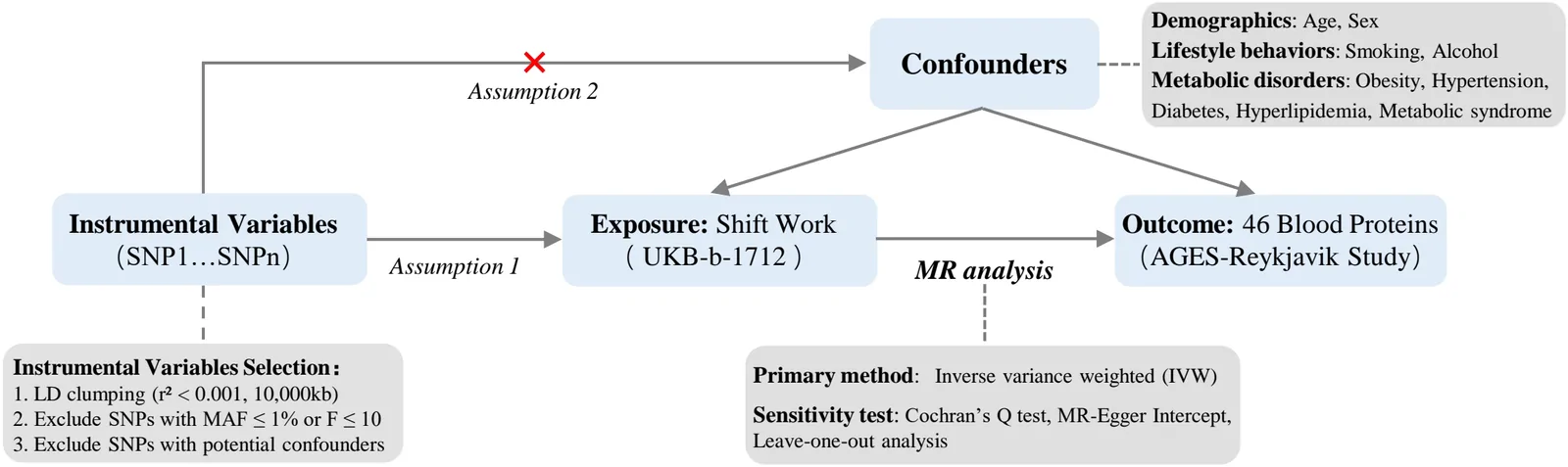
Study workflow evaluating the causal influence of shift work on 46 circulating proteins by Mendelian randomization (MR). • Assumption 2 → Confounders. Demographics: age, sex; lifestyle: smoking, alcohol; metabolic disorders: obesity, hypertension, diabetes, hyperlipidemia, metabolic syndrome. • Assumption 1 → Exposure. Shift work (UKB-b-1712) → MR analysis → Outcome. Forty-six blood proteins (AGES-Reykjavik Study).

The MR framework, summarized in **Figure 1** and aligned with the STROBE-MR checklist (17), evaluated the causal effect of shift work on 46 circulating serum proteins using summary-level genome-wide association study (GWAS) data. Single-nucleotide polymorphisms (SNPs) served as instrumental variables (IVs). For validity, each IV had to satisfy three core assumptions: (a) strong association with the exposure (shift work); (b) independence from confounders; and (c) exclusivity of the exposure–outcome pathway, acting on protein levels only through shift work. Fulfillment of these assumptions is essential for unbiased causal estimation.

#### 2.1.2 Data sources

Exposure data for shift work were obtained from the UK Biobank (ID: UKB-b-1712), which includes 263, 315 participants and 9, 851, 867 SNPs (https://gwas.mrcieu.ac.uk/) (18). Outcome data for 46 circulating proteins came from the AGES-Reykjavik Study in Iceland, a large-scale proteogenomic GWAS including 2, 091 proteins in 5, 368 individuals (https://www.ebi.ac.uk/gwas/) (19). Given the independence of the UK-based GWAS (UKB-b-1712) and Icelandic AGES-Reykjavik cohort, no participant overlap is expected. All datasets are publicly available, and ethical approvals and informed consent were obtained in the original studies. Additional dataset characteristics are summarized in **Supplementary Table 1**.

#### 2.1.3 Selection of protein outcomes

Consistent with previous genetic studies evaluating defined panels of circulating cytokines, the MR outcome set was defined as a targeted panel of cytokines and cytokine signaling proteins rather than a proteome wide survey (20, 21). The 46 protein level outcomes were manually curated from the AGES Reykjavik catalogue using published biological evidence and established immune signaling pathways. First, we selected circulating cytokines, chemokines, and immunoregulatory ligands on the basis of their established roles in inflammatory and immune regulation. Evidence that macrophage intrinsic circadian clocks regulate inflammatory cytokine production and that chronic circadian disruption alters innate immune responses provided the biological rationale for this cytokine focused approach (22, 23). Second, because immune regulation involves both intracellular cytokine signaling and ligand receptor interactions, we expanded the panel beyond conventional circulating cytokines to include selected components of JAK and STAT signaling and TGF β superfamily and SMAD signaling, together with FGL1 as an immunoregulatory ligand of LAG3 (24–29). The final panel comprised 31 interleukin designated targets, CXCL8, IFN γ, TNF α, TGF β1, FGL1, and 10 intracellular signaling proteins, yielding 46 protein level outcomes.

#### 2.1.4 Instrumental variable selection

Because validated genomic instruments that directly capture circadian rhythmicity or misalignment are currently scarce, we selected instruments from the largest available GWAS of self-reported shift-work employment (UKB-b-1712) as a pragmatic proxy for circadian disruption, acknowledging that these variants may also reflect non-circadian aspects of shift-work exposure. UKB-b-1712 phenotype is derived from a single self-reported item (UK Biobank Field 826: ‘Does your work involve shift work?’), rated on a four-level frequency scale (never/rarely, sometimes, usually, always), without differentiating night-shift status, shift type, or cumulative duration of exposure.

In MR analyses, at least 10 single-nucleotide polymorphisms (SNPs) are recommended as instrumental variables (IVs) to achieve adequate statistical power (30). Initially, SNPs were selected using the genome-wide significance threshold of P < 5 × 10^–8^. If fewer than 10 independent variants met this criterion (P < 5 × 10^-8^), the threshold was gradually relaxed in a stepwise manner (sequentially adopting thresholds of P < 1 × 10^–7^, P < 5 × 10^–7^, P < 1 × 10^–6^, P < 5 × 10^–6^, and P < 1 × 10^–5^), with an upper limit of 5 × 10^–5^, until at least 10 independent instrumental variables were obtained. The resulting SNPs then underwent the following quality-control steps (1): LD clumping (r^2^ < 0.001 within a 10–000 kb window) to ensure variant independence (2); exclusion of SNPs with a minor allele frequency ≤ 1% to minimize rare-variant bias (3); removal of weak instruments (F-statistic ≤ 10); and (4) elimination of variants associated with potential confounders-demographic factors (age, sex), lifestyle behaviors (smoking, alcohol), and metabolic traits (obesity, hypertension, diabetes, hyperlipidemia, metabolic syndrome)-as identified in the GWAS Catalog (https://www.ebi.ac.uk/gwas/). Additionnally, we performed the MR-PRESSO global test to detect outlier variants and the Steiger directionality test to confirm that the variance explained in the exposure exceeded that in the outcome.

#### 2.1.5 Statistical analysis

Five MR estimators were implemented, including random inverse-variance weighted (IVW), MR-Egger, weighted median, simple mode, and weighted mode approaches (30). IVW served as the primary estimator because it yields the most precise effect sizes when horizontal pleiotropy is absent (31). The causal associations in MR were expressed at nominal significance (P < 0.05) and false discovery rate (FDR)-adjusted significance (P_FDR_ < 0.05), and their effect sizes were expressed as β coefficients (95% CIs) for the 46 circulating proteins as continuous outcomes, and as odds ratios (ORs, 95% CIs) for shift work as a binary outcome.

**Figure 2:**
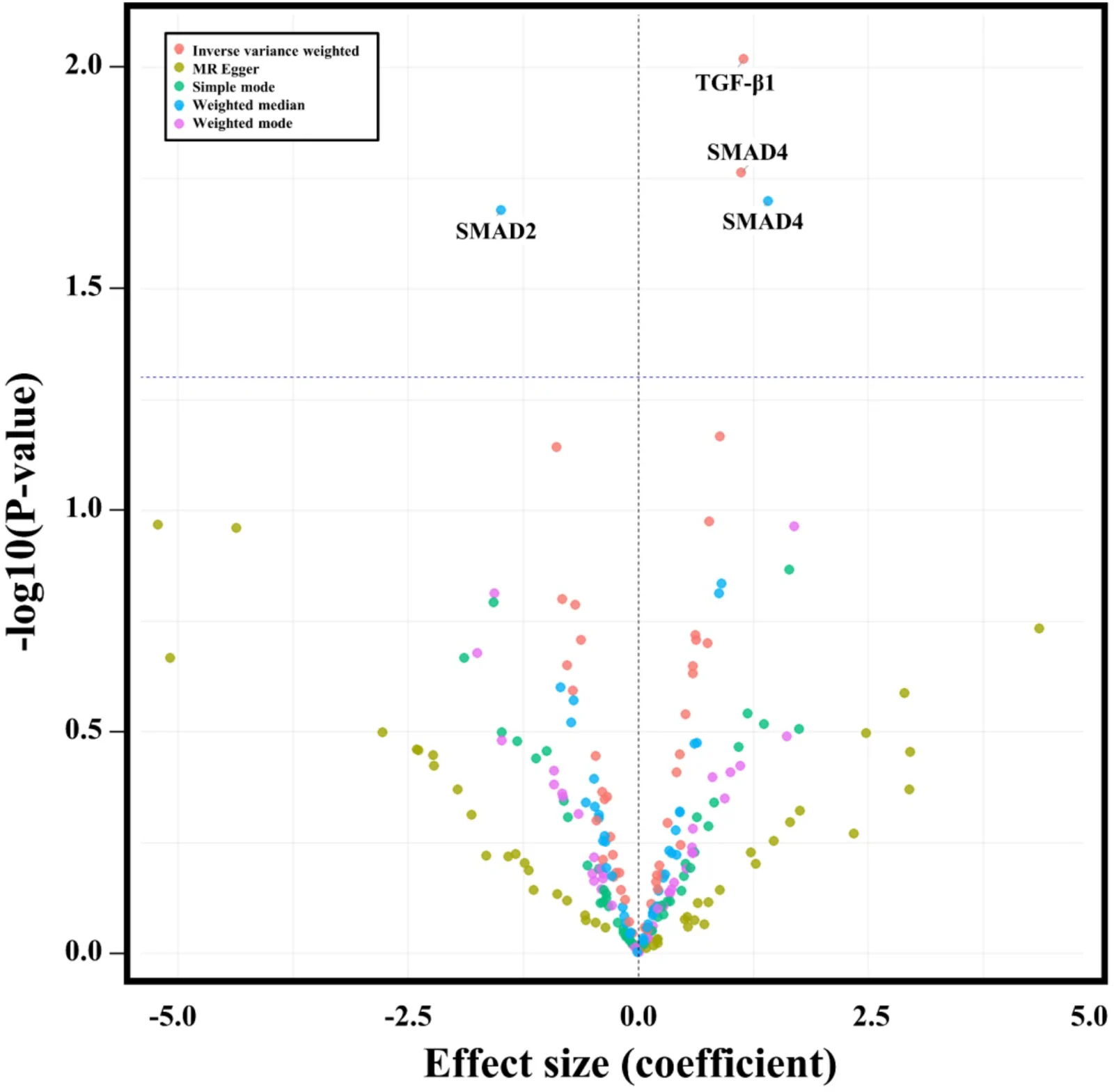
Volcano plot of immune markers associated with shift work stratified by MR method.

**Figure 3:**
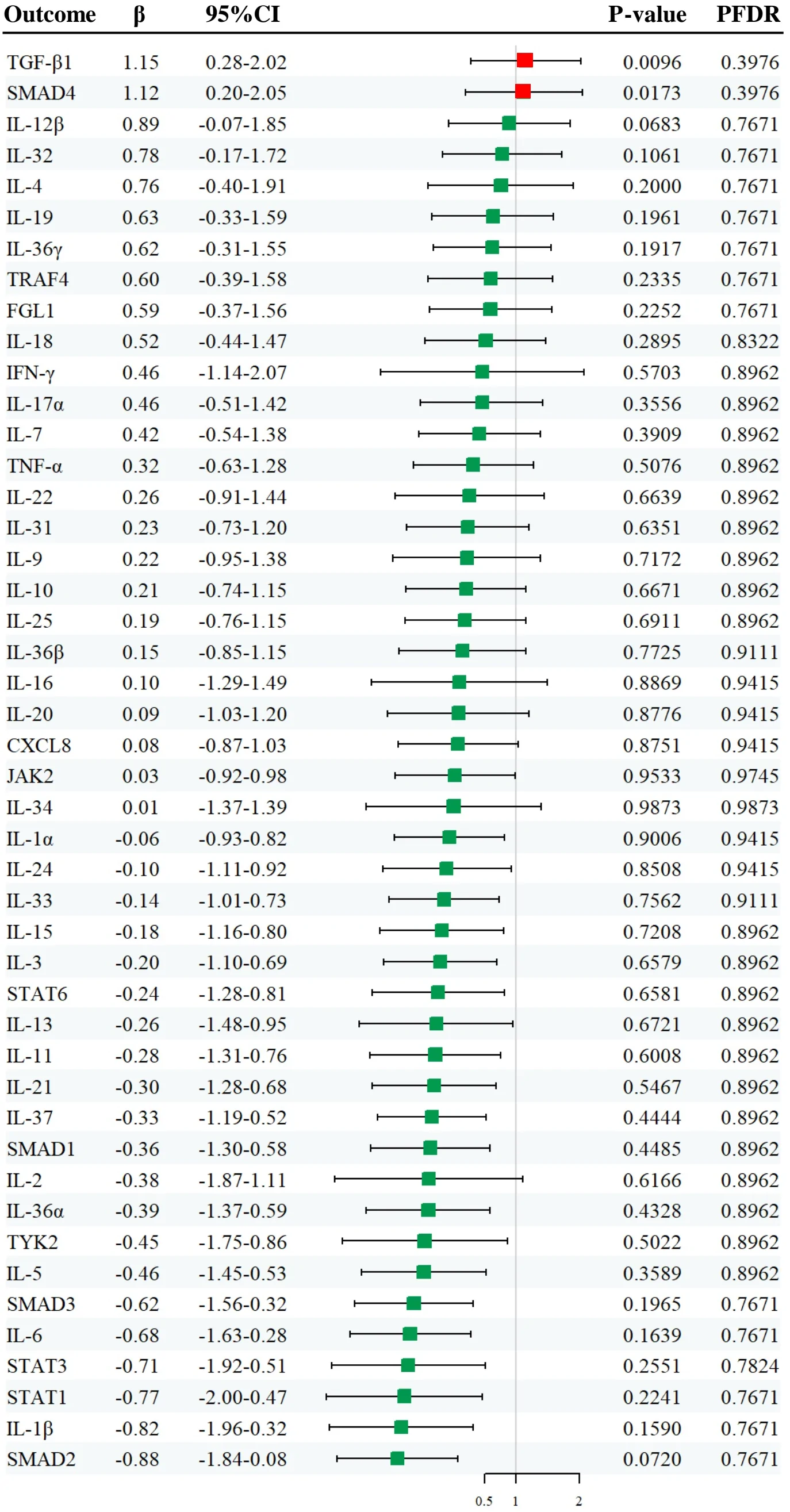
Causal effect estimates of shift work on 46 circulating proteins (IVW).

**Figure 4:**
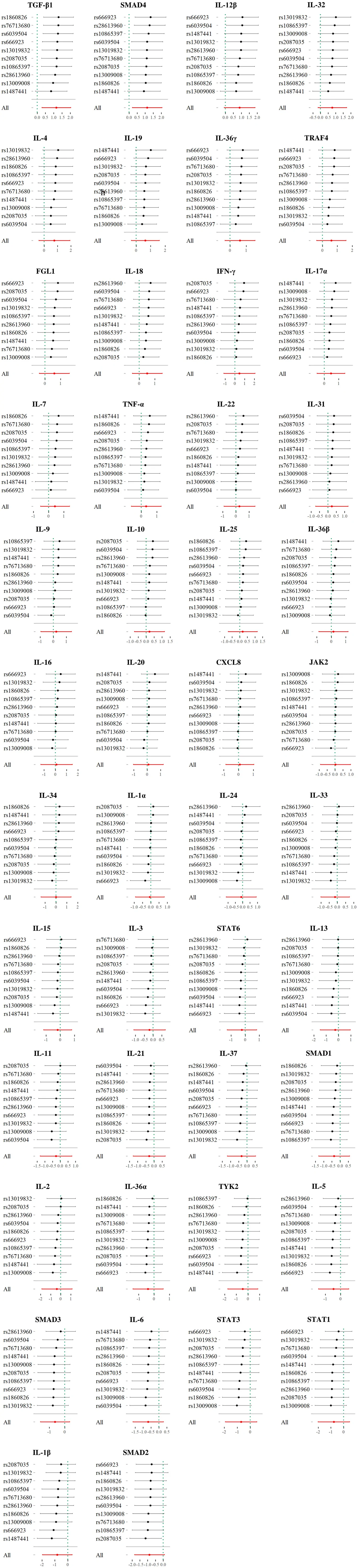
Leave-one-out sensitivity analysis for the association between shift work and each protein.

To evaluate the validity of instrumental variable assumptions, a comprehensive set of sensitivity analyses was performed. SNP heterogeneity was assessed using Cochran’s Q statistic (32). Horizontal pleiotropy was evaluated via the MR-Egger regression intercept (33), and outliers were detected using the MR-PRESSO test. Furthermore, leave-one-out analysis was conducted to examine the influence of individual SNPs (34), and reverse MR analysis was performed to exclude potential reverse causation. All statistical analyses and visualizations were performed using R, with the TwoSampleMR package (v0.6.15) applied for MR analyses. The comprehensive results of these MR analyses were visualized using a volcano plot (**Figure 2**) and forest plots (**Figures 3**, **4**).

### 2.2 Observational study

#### 2.2.1 Study participants

We conducted an observational study among medical staff from a regionally representative tertiary hospital (35). Of the 151 workers enrolled in a previous cohort (6), those who voluntarily consented to blood collection for immune-biomarker profiling were eligible. Ultimately, 61 participants contributed peripheral blood of adequate volume and quality for multiplex immunoassay. At the same visit, participants completed a structured questionnaire covering sociodemographic variables (age, sex, education) and occupational factors (current job title, work schedule, cumulative shift-work duration, weekly hours). All enrollment was voluntary and preceded by written informed consent. Trained interviewers administered the questionnaire face-to-face using a standardized script. The Institutional Review Board of Chung-Ang University approved the protocol (1041078–20231024 HR-287), and all procedures complied with the Declaration of Helsinki.

#### 2.2.2 Blood sampling

Peripheral venous blood was drawn at a single clinical site. To limit inter-day variability and potential effects of recent shifts on immune markers (36), all 61 samples were obtained on the same day before the participants’ scheduled work. Trained phlebotomists performed standardized antecubital venipuncture under sterile conditions. Approximately 10 mL of blood per participant was divided between EDTA-coated tubes (BD Vacutainer K2E, Becton Dickinson, NJ, USA) for plasma preparation and clot-activator serum-separator tubes (VACUETTE 5 mL CAT, Greiner Bio-One, Austria) for serum preparation. Following collection, the tubes were gently inverted 8–10 times. EDTA-anticoagulated blood was maintained at 4 °C and centrifuged once within 1 h of collection at 2, 263 × g for 10 min at 4 °C. The resulting plasma was aliquoted into sterile cryovials and stored at −80 °C until analysis. Serum-separator tubes were maintained upright at room temperature for 30 min to allow clot formation and were subsequently centrifuged once at 2, 263 × g for 10 min at 4 °C. The resulting serum was aliquoted into sterile cryovials and stored at −80 °C until analysis. No additional platelet-depletion centrifugation was performed. All pre-analytical procedures, including sample handling, centrifugation, aliquoting, and storage, followed a standardized operating protocol. Hemolyzed samples were excluded, and repeated freeze-thaw cycles were avoided. Sample processing was conducted in a controlled laboratory using certified refrigerated centrifuges.

#### 2.2.3 Biochemical analysis

We quantified eight cytokines to characterize shift work–related systemic inflammation: IL-1β, IL-6, IL-8, TNF-α, IFN-γ, IL-18, IL-10, and TGF-β1. TGF-β1 was included because MR supports a causal role, whereas the remaining analytes were selected on strong observational evidence of dysregulation in shift workers (10, 37, 38).

IL-1β, IL-6, IL-8, IL-10, IL-18, TNF-α, and IFN-γ were quantified with a custom 7-plex Luminex panel on a Luminex 200 system (Luminex, USA) following the manufacturer’s protocol. Plasma samples were analyzed in duplicate. Mean fluorescence intensities were converted to absolute concentrations (pg/mL) using five-parameter logistic interpolation, and R² values exceeded 0.99 for all standard curves.

Limits of detection (LODs) reported in **Supplementary Table 2**, were 0.8 pg/mL for IL-1β, 1.7 pg/mL for IL-6, 1.8 pg/mL for IL-8, 1.6 pg/mL for IL-10, 1.93 pg/mL for IL-18, 1.2 pg/mL for TNF-α, and 0.22 pg/mL for IFN-γ. Values below the LOD were considered non-detectable in the primary analysis; where imputation was required, values were drawn from a uniform distribution between LOD/2 and LOD. TGF-β1 was quantified in serum using a commercial ELISA and read on a Multiskan SkyHigh microplate reader (Thermo Fisher Scientific, USA), following the manufacturer’s protocol. The assay had a dynamic range of 15.6-1, 000 pg/mL and an LOD of 5 pg/mL. Plate blanks and standards were run analyzed in parallel to monitor assay performance.

#### 2.2.4 Statistical analysis

Analyses were performed in SAS version 9.4 (SAS Institute, Cary, NC, USA). Two-sided p-values < 0.05 were considered significant. Categorical variables are presented as counts and percentages, and continuous variables as mean ± standard error (SE). Associations between shift-work status and each cytokine concentration were evaluated using linear regression, with non-shift workers as the reference group. The crude model was unadjusted; Model 1 was adjusted for age; Model 2 for age and sex; and Model 3 for age, sex, and weekly working hours. Because no multiplicity correction was applied across the eight cytokines, P-value < 0.05 were considered nominally significant, and the findings were interpreted as exploratory.

## 3 Results

### 3.1 Two-sample MR study

#### 3.1.1 Selection and validation of SNPs

In the GWAS of shift work (UKB-b-1712), only one SNP reached the genome-wide threshold (P < 5 × 10^−8^) after linkage disequilibrium (LD) clumping (r^2^ < 0.001 within 10, 000 kb). Following the stepwise relaxation protocol described in Materials and methods, the threshold was relaxed to P < 1 × 10^−6^, identifying 11 independent SNPs (minor allele frequency > 1%, F-statistics > 10). One instrument (rs622614) was removed because of potential pleiotropy with metabolic syndrome, leaving 10 SNPs as valid IVs for the MR analysis. Detailed SNP characteristics are provided in **Table 1**. And the mapped genes and functional annotations for these 10 instrumental SNPs are summarized in **Supplementary Table 3**. Briefly, these variants are linked to shift work propensity through core circadian clock signaling, chronotype-related neuronal function, stress-responsive pathways, and sleep regulatory networks.

**Table 1:** Details of instrumental variables (IVs) selected from the GWAS of shift work.

SNP	Chr	Position	EA	OA	P-value	Beta	SE	MAF	R²	F-statistics
rs666923	1	234812041	C	A	6.50E-07	0.0141163	0.00283646	0.207135	6.55E-05	17.2356
rs13019832	2	60710571	A	G	1.40E-08	-0.0132382	0.00233128	0.415708	8.51E-05	22.4190
rs10865397	2	73358292	G	A	3.30E-07	-0.011909	0.00233352	0.476557	7.08E-05	18.6324
rs13009008	2	174043233	G	A	9.00E-07	-0.0119654	0.00243581	0.328248	6.31E-05	16.6263
rs2087035	3	131264657	C	T	1.90E-07	-0.0159674	0.00306423	0.169258	7.17E-05	18.8807
rs1487441	6	98553894	A	G	2.20E-12	-0.0161493	0.00230054	0.484375	0.000130	34.3069
rs76713680	6	105878480	T	C	4.60E-07	0.0320549	0.00635982	0.034675	6.88E-05	18.1139
rs1860826	7	2112506	A	G	7.10E-07	-0.0119023	0.00240053	0.358721	6.52E-05	17.1631
rs28613960	16	26213558	A	G	6.40E-07	-0.0114863	0.00230665	0.454628	6.54E-05	17.2282
rs6039504	20	9547230	T	C	1.60E-07	-0.0161786	0.00308805	0.163981	7.18E-05	18.8984

Chr, Chromosome; EA, Effect Allele; OA, Other Allele; SE, Standard error; MAF, Minor allele frequency.

#### 3.1.2 Causal effect of shift work

MR evaluated the impact of shift work on 46 circulating proteins. As shown in **Figure 2**, the volcano plot of MR identified nominal associations (P < 0.05) for TGF-β1, SMAD4, and SMAD2 (**Figure 2**). TGF-β1 showed a positive association in the IVW analysis, SMAD4 showed positive associations in the IVW and weighted median analyses, whereas SMAD2 showed an inverse association in the weighted median analysis. The overall causal estimates from the primary IVW analysis are visualized in **Figure 3**, with two proteins showing nominal causal associations with shift work predisposition at a threshold of P < 0.05: TGF-β1 (P = 0.0096; β=1.15, 95% CI 0.28-2.02) and its downstream effector SMAD4 (P = 0.0173; β=1.12, 95% CI 0.20-2.05), though neither remained significant after FDR correction (P_FDR_ > 0.05).

In the primary IVW analysis, genetically proxied shift-work involvement was nominally associated with TGF-β1 (β=1.15; 95% CI, 0.28-2.02; P = 0.0096) and SMAD4 (β = 1.12; 95% CI, 0.20-2.05; P = 0.0173); however, neither association remained statistically significant after FDR correction. For TGF-β1, the weighted median (β=0.88; 95% CI, -0.34-2.10; P = 0.1579), weighted mode (β=0.59; 95% CI, -1.21-2.39; P = 0.5357), and simple mode (β= 0.51; 95% CI, -1.41-2.43; P = 0.6131) estimates were not statistically significant. The MR-Egger estimate was also non-significant and was directionally opposite to the IVW estimate (β=-0.35; 95% CI, -4.65-3.94; P = 0.8767). For SMAD4, the weighted median analysis supported a nominal association in the same direction as the IVW estimate (β=1.42; 95% CI, 0.18-2.65; P = 0.0246), whereas the simple mode (β=1.64; 95% CI, -0.13-3.42; P = 0.1021), weighted mode (β=1.70; 95% CI, -0.26-3.65; P = 0.1229), and MR-Egger (β=2.48; 95% CI, -2.09-7.05; P = 0.3181) estimates were directionally concordant but not statistically significant. Overall, agreement across the five MR estimators was incomplete: TGF-β1 was supported only by IVW and showed an oppositely directed MR-Egger estimate, whereas SMAD4 showed greater directional consistency but limited statistical support. Accordingly, both associations were interpreted as exploratory rather than robust causal evidence. However, the intermediate canonical mediators SMAD2 and SMAD3 showed inverse IVW estimates (SMAD2: β=-0.88; 95% CI, -1.84-0.08; SMAD3: β=-0.62; 95% CI, -1.56-0.32). The weighted median estimate for SMAD2 was also statistically significant in the inverse direction (P = 0.022), whereas the corresponding IVW estimate was not statistically significant. Thus, the SMAD-family findings were not directionally coherent and did not support coordinated activation of the canonical TGF-β/SMAD pathway.

Sensitivity analyses for TGF-β1 and SMAD4 did not detect substantial inter-variant heterogeneity based on Cochran’s Q test, directional horizontal pleiotropy based on the MR-Egger intercept, or outlier variants based on MR-PRESSO (**Table 2**). Leave-one-out analyses indicated that the IVW estimates were not driven by a single variant (**Figure 4**). Reverse MR analyses did not provide evidence supporting an effect of TGF-β1 or SMAD4 on shift-work propensity (**Supplementary Tables 5, 6**).

**Table 2:** Results of MR sensitivity from Cochran’s Q test and MR Egger intercept test.

Outcome	IVW - Ccochran’s Q test		MR - egger intercept test		MR - PRESSO test	
	Q - statistic	P-value	Egger intercept	P-value	Global test	P-value
TGF-β1	6.7467	0.6635	0.0214	0.5037	8.5739	0.6620
SMAD4	4.7171	0.8582	-0.0193	0.5683	5.8792	0.8490
IL-12β	7.4838	0.5869	0.0317	0.3750	9.0444	0.5900
IL-32	5.4648	0.7921	-0.0125	0.7163	7.0067	0.7620
IL-4	14.0877	0.1192	-0.0313	0.4735	17.2071	0.1420
IL-19	7.2827	0.6077	0.0265	0.4539	9.3401	0.5680
IL-36γ	5.5759	0.7815	0.0368	0.2927	6.8134	0.7830
TRAF4	9.5992	0.3839	0.0343	0.3503	11.8795	0.3880
FGL1	7.3249	0.6033	0.0012	0.9733	8.8324	0.6110
IL-18	6.6294	0.6756	0.0139	0.6906	8.0748	0.6810
IFN-γ	24.8130	0.0032	0.0790	0.1723	30.3040	0.0030
IL-17α	8.5558	0.4792	-0.0045	0.9022	10.7405	0.4700
IL-7	8.9840	0.4388	0.0185	0.6122	11.0406	0.4390
TNF-α	5.4703	0.7915	-0.0082	0.8134	6.8158	0.7860
IL-22	13.6527	0.1352	0.0273	0.5408	16.4942	0.1680
IL-31	3.2022	0.9557	0.0143	0.6851	3.8777	0.9670
IL-9	12.8908	0.1676	0.0000	0.9992	15.7471	0.1900
IL-10	9.2665	0.4130	0.0345	0.3270	11.2748	0.4230
IL-25	7.4139	0.5941	-0.0183	0.6018	9.1266	0.5940
IL-36β	9.6694	0.3779	0.0643	0.0939	12.0894	0.3760
IL-16	20.4619	0.0153	-0.0320	0.5460	24.6910	0.0240
IL-20	16.5253	0.0567	-0.0075	0.8618	21.6611	0.0620
CXCL8	5.8221	0.7576	0.0350	0.3244	7.7562	0.7150
JAK2	6.0201	0.7379	-0.0409	0.2540	7.1968	0.7460
IL-34	21.1525	0.0120	-0.0418	0.4199	26.1056	0.0160
IL-1α	8.6165	0.4734	-0.0039	0.9066	10.4529	0.4730
IL-24	11.2102	0.2616	0.0066	0.8656	13.8084	0.2750
IL-33	8.1541	0.5187	-0.0194	0.5450	10.3471	0.4850
IL-15	8.8948	0.4470	-0.0208	0.5746	11.1476	0.4330
IL-3	11.1314	0.2668	0.0288	0.3906	13.4530	0.2870
STAT6	11.0369	0.2732	-0.0047	0.9071	13.4744	0.2990
IL-13	14.8767	0.0944	-0.0115	0.8052	18.3459	0.1180
IL-11	11.7159	0.2298	0.0357	0.3551	14.1673	0.2530
IL-21	1.5322	0.9969	0.0039	0.9124	1.9235	0.9980
IL-37	8.4219	0.4922	-0.0141	0.6566	10.3217	0.4860
SMAD1	5.3098	0.8065	-0.0303	0.3854	6.4306	0.8190
IL-2	20.7558	0.0138	-0.0157	0.7837	25.6743	0.0230
IL-36α	5.6283	0.7765	-0.0086	0.8096	6.7892	0.7830
TYK2	16.0833	0.0652	-0.0685	0.1413	20.4967	0.0700
IL-5	9.9114	0.3577	0.0135	0.7204	11.9661	0.3830
SMAD3	9.6192	0.3822	0.0080	0.8242	11.6605	0.4050
IL-6	3.8291	0.9223	0.0246	0.4840	4.7635	0.9270
STAT3	14.8230	0.0959	-0.0124	0.7894	17.9036	0.1220
STAT1	15.2577	0.0841	0.0634	0.1536	18.4243	0.1090
IL-1β	12.9643	0.1642	0.0044	0.9192	16.6982	0.1770
SMAD2	4.9076	0.8423	-0.0202	0.5653	6.0847	0.8380

### 3.2 Observational study

Baseline characteristics of the 61 participants are shown in **Table 3**. Overall, 27.9% worked shifts and 72.1% followed daytime schedules. Shift workers were younger, whereas sex and educational attainment did not differ significantly between the groups. Occupational composition varied: nurses constituted the largest category (57.3%) and were evenly distributed, whereas physicians were over-represented among shift workers (41.2%), and allied health or administrative staff appeared only in the non-shift group. Shift workers also had a shorter duration of employment in their current position. Most participants (73.8%) worked 40–52 h per week; nevertheless, 35.3% of shift workers reported ≥60 h per week compared with13.6% of non-shift workers, although this difference was not statistically significant.

**Table 3:** General characteristics of participants (n = 61).

Characteristic	All	Shift workers	Non-shift workers	p-value
Total participants, n (%)	61 (100.0)	17 (27.9)	44 (72.1)	
Age, years (mean ± SD)	38.4 ± 9.1	30.4 ± 2.8	41.4 ± 8.8	<0.0001
Sex, n (%)				0.7233
Male	12 (19.7)	4 (23.5)	8 (18.2)	
Female	49 (80.3)	13 (76.5)	36 (81.8)	
Education, n (%)				0.6610
High school	1 (1.6)	0 (0.0)	1 (2.3)	
College	43 (70.5)	11 (64.7)	32 (72.7)	
University graduate	17 (27.9)	6 (35.3)	11 (25.0)	
Occupation category, n (%)				0.0001
Nurses	35 (57.3)	10 (58.8)	25 (56.8)	
Other healthcare professionals	6 (9.8)	0 (0.0)	6 (13.6)	
Administrative staff	8 (13.1)	0 (0.0)	8 (18.2)	
Physicians	11 (18.0)	7 (41.2)	4 (9.1)	
Others	1 (1.6)	0 (0.0)	1 (2.3)	
Work duration, months (mean ± SD)	132.9 ± 108.8	72.0 ± 38.6	156.5 ± 118.0	0.0001
Working hours per week, n (%)				0.2054
<40 h	1 (1.6)	0 (0.0)	1 (2.3)	
40 ≤ 52 h	45 (73.8)	10 (58.8)	35 (79.5)	
52 ≤ 60 h	3 (4.9)	1 (5.9)	2 (4.5)	
≥ 60 h	12 (19.7)	6 (35.3)	6 (13.6)	

**Figure 5:**
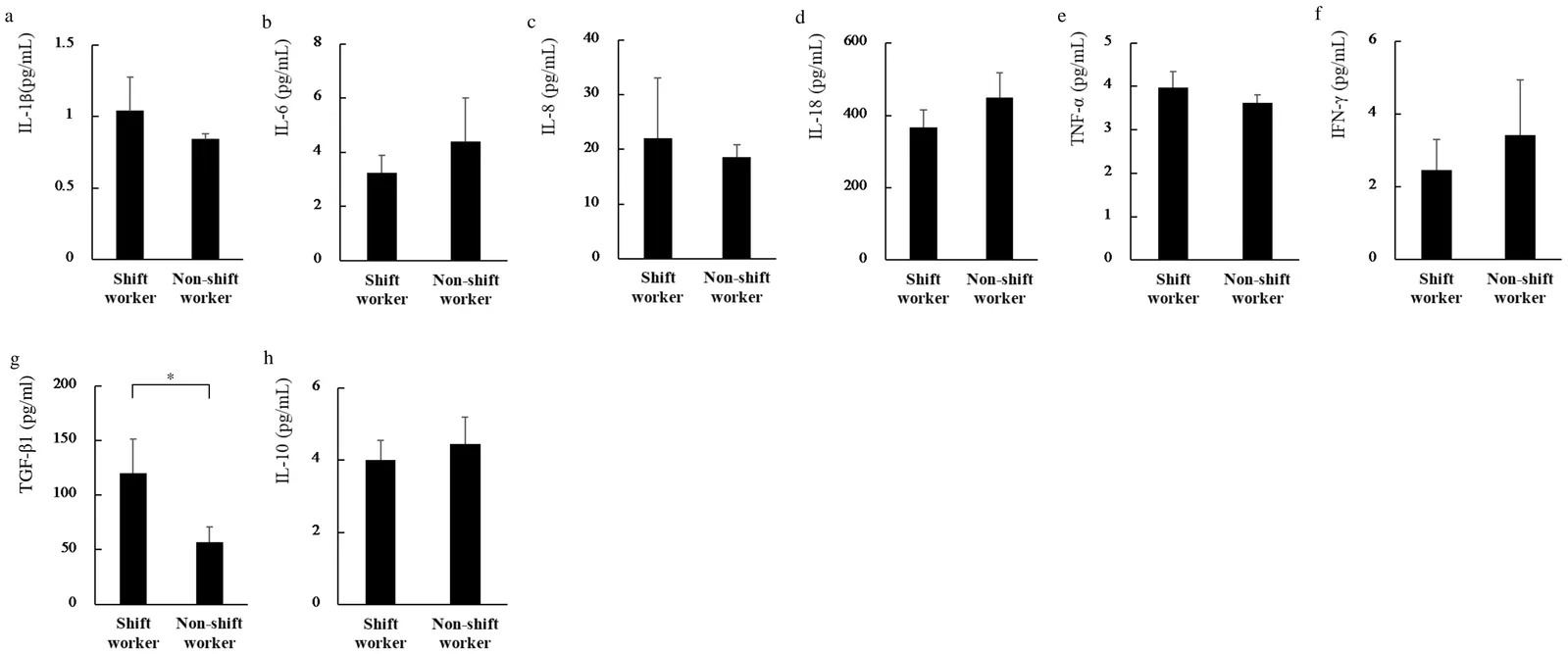
Unadjusted circulating cytokine concentrations in shift and non-shift workers. **(a)** IL-1β, **(b)** IL-6, **(c)** IL-8, **(d)** IL-18, **(e)** TNF-α, **(f)** IFN-γ, **(g)** TGF-β1, and **(h)** IL-10. Bars represent mean concentrations, and error bars indicate the standard error of the mean (SE). Statistical significance for the unadjusted between-group comparison was defined as P < 0.05 (*).

**Figure 5** compares circulating cytokine concentrations between shift and non-shift workers. IL-1β, IL-6, IL-8, IL-10, IL-18, TNF-α, and IFN-γ were measured in EDTA plasma, whereas TGF-β1 was measured in serum. In the crude linear regression analysis, shift work was nominally associated with a higher TGF-β1 concentration (β=41.7780; 95% CI, 3.5469-80.0131). However, the estimate was attenuated and was no longer statistically significant after adjustment for age (β=23.1266; 95% CI, -22.1685-68.4217). Additional adjustment for sex (β=26.2836; 95% CI, -18.5183-71.0856) and weekly working hours (β=30.8646; 95% CI, -14.0377-75.7668) produced similarly imprecise, non-significant estimates. No statistically significant associations were observed for the other seven cytokines in either the crude or adjusted models. Given the limited sample size and the absence of multiplicity correction across the eight cytokines, these observational findings were interpreted as exploratory (**Supplementary Table 7**).

## 4 Discussion

This study provides exploratory genetic evidence that liability to shift work, used as a proxy for long-term exposure to nonstandard work schedules and circadian disruption, may be associated with circulating TGF-β1 and SMAD4 signals. However, neither association survived FDR correction, and the discordant SMAD2/3 estimates did not support coherent canonical TGF-β/SMAD pathway activation. The observational analysis showed a directionally consistent but non-significant age-adjusted association and should therefore be considered supportive and exploratory rather than confirmatory evidence.

TGF-β1 is a context-dependent immunoregulatory cytokine involved in immune regulation, tissue remodeling, and redox homeostasis. Experimental studies suggest that circadian misalignment can suppress BMAL1-NRF2 antioxidant pathways, allowing reactive oxygen species to activate latent TGF-β1 and remodel chromatin at the TGFB1 locus, thereby amplifying its expression (39, 40). Dysregulated TGF-β1 has been implicated in cardiometabolic dysfunction, pulmonary fibrosis, steatohepatitis, vascular remodeling, and tumor progression, although its biological effects are highly context dependent all conditions reported at higher prevalence among shift workers (41–43).

Numerous epidemiological evidence have linked shift work to higher rates of obesity, metabolic syndrome, hypertension, cardiovascular disease, and several malignancies, notably breast and gastric cancers (44). Circadian misalignment-induced TGF-β1 elevation may represent a plausible common mechanism. In adipose tissue, TGF-β1 appears to drive fibrosis and inflammation, potentially impairing insulin sensitivity and promoting metabolic disease (45). Within the vasculature and myocardium, excessive TGF-β1 could triggers pathological remodeling and myocardial fibrosis, thereby fostering hypertension and accelerating cardiovascular disease (46, 47). Elevated TGF-β1 might also conditions the tumor microenvironment; in breast tissue, it may promote epithelial–mesenchymal transition (EMT) and immune evasion, whereas in the stomach it could activate cancer-associated fibroblasts and suppresses immune surveillance, collectively enhancing tumor growth and metastasis (48). Despite these epidemiological founding, studies investigating systemic inflammation in shift workers remains inconclusive. While some investigations reported elevated concentrations of IL-6, IL-1β, IL-10, and TNF-α, large cohorts observed no differences in these or related markers, including IFN-γ (8, 11, 49). Similarly, a recent comprehensive analysis found that neither night-shift status nor short sleep duration significantly altered immune indices (38). These discrepancies likely underscore the limitations of observational designs, where unmeasured confounding factors and reverse causation can obscure true causal associations (50).

To address these challenges, we implemented a MR strategy to strengthen causal inference regarding the association between shift-work liability and circulating immune biomarkers. Under its underlying assumptions, MR is less susceptible to residual confounding and reverse causation than conventional observational analyses (51). The complementary observational analysis provided directionally supportive, although non-confirmatory, evidence for the TGF-β1 signal.

Given that shift work accounts for nearly one in five employees worldwide and about 10% of the Korean workforce, and imposes an annual economic burden exceeding US$160 billion, our findings could suggest significant public health implications (52, 53). Particularly in light of the International Agency for Research on Cancer (IARC) classification of night-shift work as “probably carcinogenic to humans” (Group 2A), our identification of TGF-β1 as a potential biomarker supports a need for targeted occupational interventions including biomonitoring programs and preventive health policies (54).

Despite the large-scale GWAS datasets used in the MR analysis, several limitations should be considered. First, although horizontal pleiotropy was assessed using MR-Egger and MR-PRESSO, residual pleiotropic bias cannot be excluded. Second, conventional MR estimates may not fully capture nonlinear effects or gene-environment interactions, potentially oversimplifying the heterogeneous effects of shift-work exposure. Third, because the MR analyses were based predominantly on European-ancestry datasets, the findings should be replicated in more diverse populations. Moreover, the genetic instruments captured only selected aspects of shift work and did not fully represent schedule type, intensity, duration, or individual tolerance. Future studies should refine shift-work phenotypes and identify more specific genetic proxies. Fourth, the nominal associations of TGF β1 and SMAD4 did not remain significant after FDR correction, and the inverse estimates observed for SMAD2 and SMAD3 did not support a consistent pattern across the TGF β and SMAD signaling pathway. These findings should therefore be interpreted as exploratory protein specific associations rather than evidence of a definitive causal pathway. Finally, the 46 protein outcomes were selected through manual curation based on the literature and established immune signaling pathways rather than through a formal ontology-based procedure. The panel may therefore not capture all immune related proteins available in the AGES Reykjavik resource, and the findings should be interpreted as a targeted exploratory assessment. In addition, SOMAmer signals for intracellular proteins, including JAK, STAT, and SMAD family members, represent aptamer-based signals detected in serum and should not be interpreted as direct measures of intracellular protein abundance or pathway activation.

Some limitations of the observational study warrant consideration. First, serum TGF-β1 concentrations may have been influenced by platelet release during clotting, and platelet counts or activation markers were unavailable. Although biospecimen processing was standardized protocols, residual pre-analytical variability cannot be excluded. Second, the cross-sectional design precluded assessment of temporal sequence, and several important potential confounders, including body mass index, smoking, alcohol consumption, and sleep duration, were unavailable. These limitations restricted evaluation of whether TGF-β1 alterations precede or persist with shift-work exposure and of the independent association between shift work and circulating TGF-β1. Third, the observational analysis included only 61 participants, including 17 shift workers, and evaluated a targeted panel of eight immune mediators in healthcare workers from a single tertiary hospital. These features limited statistical power, restricted characterization of the broader immune landscape, and reduced generalizability to other occupations and shift-work patterns.

Fourth, the UK Biobank exposure was based on a single self-reported item on current shift-work involvement and did not distinguish night versus non-night shifts or cumulative exposure duration. In contrast, the observational cohort used a structured questionnaire assessing current shift pattern and cumulative duration; therefore, the MR exposure and observational exposure are not fully equivalent, and this should be considered when interpreting their convergence. Moreover, the genetic instruments primarily represent liability to engage in shift work, encompassing occupational selection, work organization, and individual tolerance to nonstandard schedules, rather than circadian misalignment itself. Although circadian disruption is an expected consequence of such schedules, the instruments do not separately capture the timing, intensity, or chronicity of circadian misalignment. Future MR studies should use more specific genetic instruments for circadian rhythmicity and misalignment to distinguish circadian-specific effects from broader shift-work-related factors. Finally, the observational analysis evaluated a targeted panel of eight immune mediators selected on the basis of previous shift-work studies (8–11, 38, 49). The limited sample size and targeted biomarker coverage reduced statistical power and did not capture the broader immune landscape; therefore, these findings should be interpreted as exploratory, hypothesis-generating support rather than definitive confirmation. Larger studies using unbiased proteomics or single-cell transcriptomic approaches are needed to characterize shift work-related immune alterations more comprehensively.

## 5 Conclusion

Integrating two-sample MR with an exploratory observational data, we identified circulating TGF-β1 as a candidate immunological signal associated with genetic liability to shift work. However, the absence of FDR-adjusted significance, discordant SMAD2/3 estimates, and attenuation of the observational TGF-β1 association after age adjustment preclude a definitive causal or canonical pathway interpretation. Independent replication in larger longitudinal cohorts, together with multi-omics analyses and direct intracellular assessment of TGF-β/SMAD activity, is required to clarify the biological and mechanistic relevance of these findings and their potential implications for preventive strategies in shift workers.

## Data Availability

The datasets presented in this study can be found in online repositories. The names of the repository/repositories and accession number(s) can be found in the article/**Supplementary Material**.
