# Supplementary material for: Circadian disruption and circulating TGF-β1 in workers: evidence from Mendelian randomization and observational data
Source: Front Immunol. 2026 Sep 10;17:1904019. doi: 10.3389/fimmu.2026.1904019 (PMC13600836; doi:10.3389/fimmu.2026.1904019)
Supplement: Supplementary file 1 [file Table7.docx]

| **Cytokine** | **β (95% confidence interval)** | | | |
| --- | --- | --- | --- | --- |
|  | **Crude** | **Model 1** | **Model 2** | **Model 3** |
| **IL-1β** | 0.1985 (−0.1198, 0.5167) | 0.1390 (-0.2442, 0.5222) | 0.1245 (-0.2601, 0.5099) | 0.1588 (-0.2292, 0.5467) |
| **IL-6** | -0.2534 (-1.0841, 0.5774) | -0.1567 (-1.1588, 0.8455) | -0.1931 (-1.2016, 0.8154) | -0.1679 (-1.1955, 0.8597) |
| **IL-8** | 3.4209 (-12.0264, 18.8683) | 4.6933 (-13.9492, 23.3358) | 3.9072 (-14.8132, 22.6275) | 4.9671 (-14.0261, 23.9603) |
| **IL-18** | -82.7640 (-309.2431, 143.7151) | 90.8349 (-169.7180, 351.3877 | 99.6321 (-162.8147, 362.0789) | 87.4889 (-179.2556, 354.2333) |
| **TNF-α** | 0.2806 (-0.51325, 1.0745 | 0.1071 (-0.8479, 1.0621) | 0.0913 (-0.8746, 1.0572) | 0.0610 (-0.9226, 1.0446) |
| **IFN-γ** | -0.6472 (-5.2092, 3.9148) | -1.7629 (-7.2455, 3.7196) | -2.0119 (-7.5098, 3.4860) | -1.7368 (-7.3214, 3.8478) |
| **IL-10** | -0.1774 (-1.4743, 1.1195) | -0.7873 (-2.3258, 0.7513) | -0.8542 (-2.3983, 0.6899) | -0.8276 (-2.4018, 0.7466) |
| **TGF-β1** | 41.7780 (3.5469, 80.0131)* | 23.1266 (-22.1685, 68.4217) | 26.2836 (-18.5183, 71.0856) | 30.8646 (-14.0377, 75.7668) |

**Supplementary Table 7. Crude and adjusted associations between shift-work status and circulating cytokine concentrations**

Model 1 adjusted age.

Model 2 adjusted age and sex.

Model 3 adjusted age, sex and working hours.

* <0.05
